# Prognostically Optimal Heart Rate at Discharge in Hospitalized Patients With Heart Failure and Atrial Fibrillation

**DOI:** 10.1016/j.jacadv.2024.101120

**Published:** 2024-07-24

**Authors:** Makoto Kishihara, Ryoko Kawakami, Noritoshi Fukushima, Takuro Abe, Takuma Takada, Shota Shirotani, Ayano Yoshida, Takehiro Hata, Shonosuke Watanabe, Takanori Kawamoto, Shun Hasegawa, Junichi Yamaguchi, Kentaro Jujo

**Affiliations:** a Department of Cardiology, Saiseikai Kazo Hospital, Saitama, Japan; b Physical Fitness Research Institute, Meiji Yasuda Life Foundation of Health and Welfare, Tokyo, Japan; c Department of Preventive Medicine and Public Health, Tokyo Medical University, Tokyo, Japan; d Department of Cardiology, Saitama Medical Center, Saitama, Japan; e Department of Cardiology, Tokyo Women’s Medical University, Tokyo, Japan; f Department of Cardiology, Tokyo Metropolitan Tama Medical Center, Tokyo, Japan; g Department of Cardiology, Kindai University Hospital, Osaka, Japan; h Department of Cardiology, Tokyo Women’s Medical University Yachiyo Medical Center, Chiba, Japan

**Keywords:** atrial fibrillation, heart failure, heart rate

## Abstract

**Background:**

Managing heart rate (HR) is crucial for enhancing clinical prognosis in patients with heart failure (HF) and atrial fibrillation (AF). Nevertheless, the prognostic impact of HR at discharge in hospitalized HF patients remains unclear.

**Objectives:**

This study aimed to determine the HR associated with the lowest risk of death and HF in patients hospitalized with HF and AF.

**Methods:**

In this observational study, 334 persistent AF patients were analyzed from a database of 1,930 consecutive HF hospitalizations. Exclusion criteria included sinus rhythm or paroxysmal AF, cardiac pacemakers, or unrecorded HR at discharge. Participants were divided into four groups based on HR at discharge in 10 beats/min increments. The primary endpoint was a composite of death from any cause and rehospitalization due to HF. The association between resting HR and the primary endpoint was determined using Kaplan-Meier analysis and Cox proportional hazards models.

**Results:**

The median follow-up period was 389 days, with 133 patients (39.8%) reaching the primary endpoint. Kaplan-Meier analysis revealed a significantly higher primary endpoint incidence in patients with HR >81 beats/min at discharge compared to those with HR <60 beats/min (log-rank test for trend: *P* = 0.039). Multivariable Cox regression analysis showed that HR >81 beats/min at discharge was associated with the primary endpoint, with a hazard ratio of 1.79 (95% CI: 1.04-3.07), compared to HR <60 beats/min.

**Conclusions:**

The findings suggest that controlling HR to less than 80 beats/min at discharge may lead to better clinical outcomes in patients with HF and persistent AF.

Heart failure (HF) is a major cause of mortality, and the number of affected patients continues to rise.^1^ For those treated with guideline-directed medications, heart rate (HR) reduction is a key strategy to improve clinical prognosis, regardless of heart rhythm.^2^^,^^3^ HF is often accompanied by various comorbidities, with atrial fibrillation (AF) being a common coexisting condition.^4^^,^^5^ Indeed, patients with HF are more likely to develop AF than the general population.^6^ A previous study found comparable clinical outcomes between strict (resting HR <80 beats/min) and lenient (resting HR <110 beats/min) HR controls in AF patients.^7^ However, this study primarily included outpatients and only a small proportion of patients with HF.^7^ Although AF-induced tachycardia can lead to HF decompensation and hospitalization, guidelines have not provided a specific target HR for patients with HF and persistent AF.^8^^,^^9^ While previous studies have explored HR control in patients with HF and AF, the optimal target HR remains unclear,^2^ and data on hospitalized patients with HF and AF are limited. Consequently, we conducted this study to identify the target HR at discharge associated with the best prognosis in patients with HF and AF.

## Methods

### Study population and endpoints

This observational study initially included consecutive patients hospitalized for HF at Tokyo Women's Medical University Hospital from July 2013 to September 2019. Cardiologists diagnosed patients with HF based on the Framingham HF diagnostic criteria.^10^ Exclusion criteria were sinus rhythm, cardiac pacemakers or other antiarrhythmic devices, paroxysmal AF, and unrecorded HR at admission or discharge. Persistent AF was defined as continuous AF sustained for more than 7 days.^9^ This study specifically included patients who exhibited persistent AF for the entire duration of their hospital stay; cases transitioning to sinus rhythm during hospitalization were not considered. The study population was classified into four groups at 10 beats/min increments of HR at discharge: HR <60 beats/min, HR ≤60 to <70 beats/min, HR ≤71 to <80 beats/min, and HR ≤81 beats/min. The primary endpoint was a composite of death from any cause and rehospitalization due to HF. This study adhered to the principles of the Declaration of Helsinki, and the Tokyo Women's Medical University ethics committee approved the study protocol (No. 2020-0028). Informed consent was obtained in the form of an opt-out due to the retrospective design.

### Data collection and follow-up

The following patient characteristics at discharge were included: age, sex, comorbidities, laboratory data (estimated glomerular filtration rate [eGFR], B-type natriuretic peptide [BNP]), echocardiographic parameters including left atrial dimension and left ventricular ejection fraction [LVEF], and oral medication use. Blood pressure was obtained using an automated oscillometric blood pressure cuff, and resting HR was assessed by health care professionals at each visit. After discharge, outpatient follow-up visits were scheduled at least every 2 months, as well as according to patients' medical needs, and patients were contacted by telephone if they missed a scheduled clinic visit.

### Statistical analyses

Continuous variables are expressed as median (IQR) and categorical variables as percentages. Patient characteristics were compared according to HR category using Kruskal-Wallis tests for continuous variables and chi-squared tests for categorical variables. The Kaplan-Meier curve within 1,000 days was used to visually summarize time-to-event data, with differences among HR categories evaluated using the log-rank test for trend. The association between resting HR and the primary endpoint was investigated using Cox proportional hazards models. Hazard ratios adjusted for age and sex with 95% CIs were calculated according to resting HR categories (model 1). In model 2, we additionally adjusted for LVEF, BNP, eGFR, beta-blocker medication, and renin-angiotensin system inhibitors (RASi) medication. Furthermore, in model 3, we additionally adjusted for body mass index (BMI), systolic blood pressure, and diabetes. To evaluate the shape of the association, we assessed nonlinear trends between resting HR and the primary endpoint using a restricted cubic spline in a Cox proportional hazards model with 3 knots placed at the 10th, 50th, and 90th percentiles of participant HR (beats/min), including the same covariates as in model 3. Several sensitivity analyses were performed to confirm the robustness of the main results. Subgroup analyses were conducted for beta-blocker medication usage and LVEF to assess potential effect modification. An analysis was also performed after excluding individuals who experienced the primary endpoint during the initial 30-day follow-up to minimize reverse causation possibilities. A 2-tailed *P* < 0.05 was considered statistically significant. Statistical analyses were performed using Stata version 17.0 (StataCorp) and SPSS Statistics version 29.0 (IBM Corp).

## Results

### Study sample

The flow chart detailing the selection process for eligible patients is presented in Supplemental Figure 1. Out of the 1,930 consecutive patients hospitalized for HF, a total of 334 were ultimately included in the analysis. Baseline characteristics for the study sample are presented in Table 1. The median age of the patients was 78 years, with men comprising 60% of the cohort. The median HR was recorded at 70 beats/min, while the median LVEF stood at 47%. The median level of BNP was 257 pg/ml, and the median eGFR was 42.3 ml/min/1.73 m^2^. A significant proportion of 82% of the patients presented with an eGFR below 60 ml/min/1.73 m^2^. Hypertension emerged as the most prevalent comorbidity, affecting 70% of the study population. Upon discharge, the prescription rates for beta-blockers and RASi were 76% and 79%, respectively.

**Table 1:** Baseline Characteristics of Patients at Discharge

	Overall (N = 334)	Heart Rate, beats/min				*P* Value
		≤60 (n = 79)	61-70 (n = 89)	71-80 (n = 101)	≥81 (n = 65)	
Age, y	78 (72-83)	78 (70-83)	78 (72-83)	78 (72-84)	78 (70-83)	0.921
Men, %	59.6	63.3	66.3	51.5	58.5	0.179
BMI, kg/m^2^^a^	21.3 (19.0-24.1)	21.3 (19.3-24.2)	21.2 (18.9-23.6)	21.9 (19.2-25.5)	20.5 (18.2-23.9)	0.190
Systolic blood pressure, mm Hg	110 (102-122)	112 (104-124)	110 (102-120)	110 (102-122)	108 (99-121)	0.280
Diastolic blood pressure, mm Hg	60 (54-64)	60 (54-64)	60 (54-64)	60 (56-70)	60 (54-64)	0.319
Heart rate, beats/min	70 (61-78)	56 (51-60)	66 (63-69)	75 (72-78)	88 (83-99)	<0.001
LVEF, %	47 (37-53)	48 (37-52)	45 (34-51)	49 (40-54)	47 (36-53)	0.048
LAD^b^, mm	51 (44-58)	53 (46-60)	52 (45-59)	50 (44-58)	50 (42-58)	0.170
Hemoglobin, g/dl	11.8 (10.4-13.4)	12.1 (10.7-13.6)	11.8 (10.5-13.8)	11.8 (10.3-13.3)	11.2 (10.0-13.2)	0.401
BNP, pg/ml	257.0 (130.5-422.8)	231.2 (96.9-391.0)	245.2 (130.5-460.6)	264.9 (136.1-430.6)	330.1 (142.6-463.8)	0.111
eGFR, ml/min/1.73 m^2^	42.3 (30.2-55.2)	43.3 (33.6-54.7)	39.4 (28.1-53.4)	43.9 (31.1-55.8)	40.3 (29.7-55.9)	0.517
Hypertension, %	69.5	70.9	73.0	68.3	64.6	0.707
Diabetes, %	36.5	31.6	39.3	39.6	33.8	0.631
Dyslipidemia, %	47.0	38.0	55.1	49.5	43.1	0.135
Medication						
Beta-blocker, %	76.3	65.8	75.3	83.2	80.0	0.046
RASi, %	79.3	79.7	80.9	80.2	75.4	0.848
Calcium channel blocker, %	27.2	29.1	30.3	27.7	20.0	0.512
MRA, %	59.3	55.7	57.3	63.4	60.0	0.735
Amiodarone, %	6.9	8.9	6.7	5.9	6.2	0.878
OAC, %	90.7	92.4	89.9	92.1	87.7	0.737

Values are median (IQR) or %.

BMI = body mass index; BNP = brain natriuretic peptide; eGFR = estimated glomerular filtration rate; LAD = left atrial dimension; LVEF = left ventricle ejection fraction; MRA = mineralocorticoid receptor antagonist; OAC = oral anticoagulant; RASi = renin-angiotensin-aldosterone system inhibitor.

a The number of overall patients was 318.

b The number of overall patients was 312.

### Clinical outcomes

**Figure 1:**
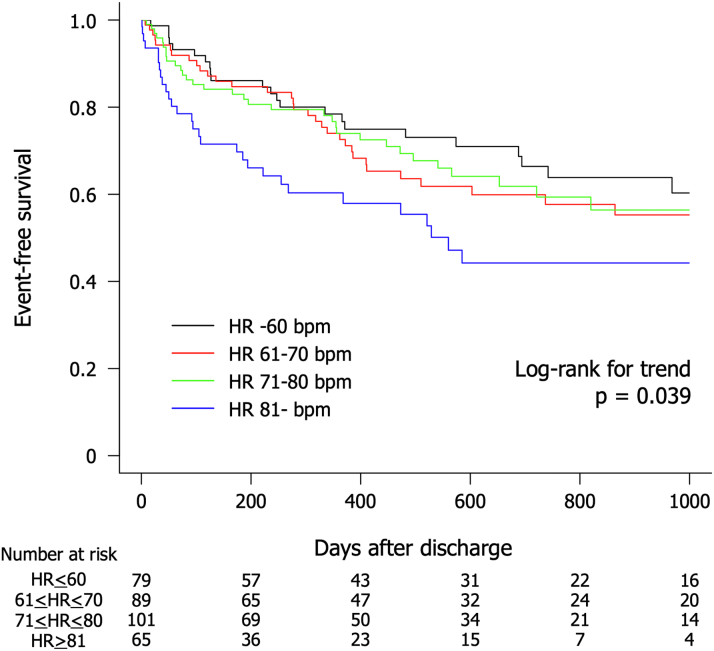
**Kaplan-Meier for the Composite Outcome According to Resting Heart Rate Category** The outcome was a composite of death from any cause and rehospitalization for heart failure. HR = heart rate.

During the observation period, which had a median follow-up of 389 days, a total of 133 patients (39.8%) experienced the composite endpoint of death from any cause and rehospitalization due to HF. The endpoint occurred in 35.4% of patients with HR <60 beats/min, 43.8% with HR ≤ 61 to <70 beats/min, 35.6% with HR ≤ 71 to <80 beats/min, and 46.2% with HR ≤81 beats/min. Kaplan-Meier analysis revealed that the group with HR at discharge ≥81 beats/min had a higher rate of the primary endpoint (log-rank test for trend: *P* = 0.039) (Figure 1). After adjusting for age, sex, LVEF, BNP, eGFR, beta-blockers medication, and RASi medication (model 2), multivariable Cox regression analysis indicated that a HR >81 beats/min at discharge was an independent predictor for the composite endpoint (hazard ratio: 1.87; 95% CI: 1.11-3.17) (Table 2). Additionally, after adjusting for BMI, systolic blood pressure, and diabetes (model 3), the association was still significant (hazard ratio: 1.79; 95% CI: 1.04-3.07) (Table 2). Central Illustration displays the restricted cubic spline of the association between resting HR and the composite endpoint of death from any cause and rehospitalization due to HF. The model was adjusted for age, sex, LVEF, BNP, eGFR, beta-blocker medication, RASi medication, BMI, systolic blood pressure, and diabetes. The solid line represents HRs, and the dashed lines represent 95% CIs for the trend obtained from restricted cubic spline regression. A reference HR of 61 beats/min was established for comparison. The restricted cubic spline analysis elucidates that a higher discharge HR correlates with a poorer prognosis.

**Table 2:** Hazard Ratios of All-Cause Mortality and Hospitalization for Heart Failure According to Heart Rate Levels

	Heart Rate, beats/min			
	≤60 (n = 79)	61-70 (n = 89)	71-80 (n = 101)	≥81 (n = 65)
Person-days	48,004	52,797	52,167	22,976
Cases	28	39	36	30
Rate/100,000 person-days	58	74	69	131
Model 1	1.00 (reference)	1.27 (0.78-2.06)	1.17 (0.71-1.93)	1.98 (1.17-3.33)
Model 2	1.00 (reference)	1.26 (0.77-2.07)	1.26 (0.76-2.08)	1.87 (1.11-3.17)
Model 3^a^	1.00 (reference)	1.18 (0.71-1.95)	1.10 (0.66-1.83)	1.79 (1.04-3.07)

Model 1: adjusted for age and sex.

Model 2: adjusted for model 1 covariates plus LVEF, BNP, eGFR, beta-blocker medication, and RASi medication.

Model 3: adjusted for model 2 covariates plus BMI, systolic blood pressure, and diabetes.

The E-value (model 1) of the hazard ratio for the ≥81 beats/min group was 3.37. The E-value (model 2) of the hazard ratio for the ≥81 beats/min group was 3.15.

The E-value (model 3) of the hazard ratio for the ≥81 beats/min group was 2.98.

BMI = body mass index; BNP = brain natriuretic peptide; eGFR = estimated glomerular filtration rate; HR = heart rate; LVEF = left ventricle ejection fraction; RASi = renin-angiotensin-aldosterone system inhibitor.

a The number of overall patients was 318.

**Central Illustration:**
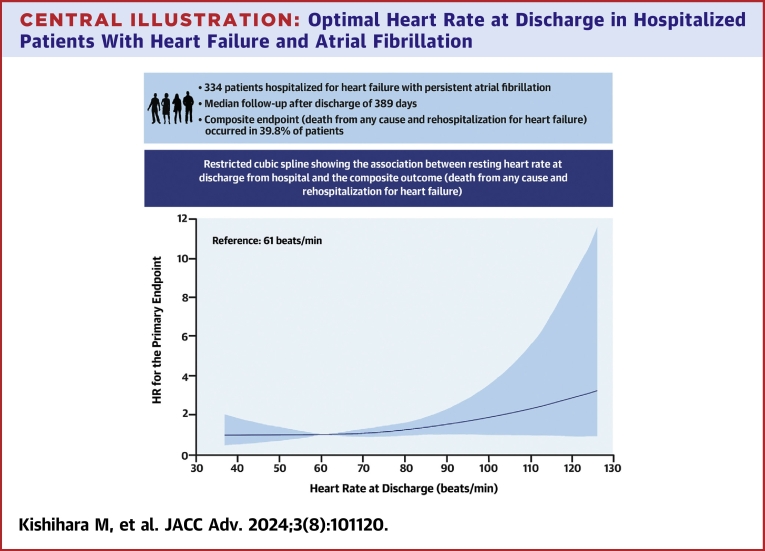
**Optimal Heart Rate at Discharge in Hospitalized Patients With Heart Failure and Atrial Fibrillation** The model was adjusted for age, sex, BMI, systolic blood pressure, diabetes, LVEF, BNP, eGFR, beta-blocker medication, and RASi medication. The solid line represents hazard ratios, and the shaded region represents 95% CIs for the trend obtained from restricted cubic spline regression (3 knots at 55, 70, and 88 beats/min). The reference value was set at 61 beats/min. Study population: 60% men. Median age: 78 years. Median follow-up period of 389 days. Conclusion: Heart rate less than 80 beats/min at discharge leads to better clinical outcomes in hospitalized patients with heart failure and persistent atrial fibrillation. BMI = body mass index; BNP = brain natriuretic peptide; eGFR = estimated glomerular filtration rate; LVEF = left ventricular ejection fraction; RASi = renin-angiotensin system inhibitor.

Supplemental Table 1 presents hazard ratios of the primary endpoint according to HR levels, specifically among beta-blocker medication users. Multivariable Cox regression analysis revealed that HR >81 beats/min at discharge remained an independent predictor for the primary endpoint in that population (hazard ratio: 2.26; 95% CI: 1.19-4.27). Supplemental Tables 2 and 3 display hazard ratios for the primary endpoint after excluding patients who experienced endpoints during the initial 30-day follow-up and hazard ratios in the subgroup analyses by LVEF, respectively. Supplemental Table 2 demonstrates that HR >81 beats/min at discharge is a predictor of death from any cause and rehospitalization for HF (hazard ratio: 1.74; 95% CI: 0.99-3.07). Supplemental Table 3 focuses on subgroup analyses based on LVEF, delineating hazard ratios for the primary endpoint. This analysis suggests a trend toward no significant difference in outcomes between patients with HF with reduced ejection fraction (HFrEF, LVEF <50%) and HF with preserved ejection fraction (HFpEF, LVEF ≥50%) when stratified by resting HR (hazard ratio: 1.60 and 1.67; 95% CI: 0.79-3.24 and 0.64-4.34, respectively). The individual components of the primary endpoint are summarized in Supplemental Table 4. An HR >81 beats/min at discharge is a significant predictor for death from any cause, but not for rehospitalization due to HF.

## Discussion

The principal finding of our study was the identification of a target HR during hospitalization that was associated with a favorable prognosis in patients with HF and AF. The prognostically optimal HR at discharge was determined to be <80 beats/min, providing greater clinical benefits for patients with HF and AF. This finding was consistent across subgroups, including patients with HFrEF and HFpEF, those receiving beta-blockers, and those experiencing outcomes during the initial 30-day follow-up. Additionally, the primary finding maintained its trend even as covariates were incrementally added and multivariate analysis was conducted. Although this study was an observational, single-center registry, it emphasizes the importance of an optimal target HR at discharge. A noteworthy aspect of our findings is the differential impact on the components of the primary endpoint. Specifically, death from any cause emerged as a more significant contributor to the overall prognosis compared to the rehospitalization due to HF. This differential impact emphasizes the need for a nuanced understanding of the role of HR management in patients with HF and AF, particularly during the acute phase. The insights gained from this study about HR management in such patients provide a valuable contribution to the field, offering a new perspective on the management of this complex patient population.

HF and AF frequently coexist, with AF occurring in more than half of patients with HF, and they can exacerbate each other.^4^ Moreover, AF increases mortality in patients with HF.^4^ The importance of HR control in AF was demonstrated in the AFFIRM and RACE-II trials.^7^^,^^11^ However, these studies did not focus specifically on patients with HF and AF, including only 10 to 20% of patients with HF.^7^^,^^11^ A notable distinction of this study, compared to previous research, lies in the patient setting. While prior studies predominantly concentrated on outpatients with AF, our research specifically targeted a hospitalized cohort. This focus on hospitalized patients provides unique insights into the management of HR in a more acute setting, where HF and AF often intersect with increased complexity and severity. This distinction underlines the importance of tailoring HR management strategies to the specific needs of hospitalized patients with both HF and AF, recognizing the nuanced challenges and implications of managing these intertwined conditions in an inpatient environment. No optimal HR at discharge had been established for better prognosis in hospitalized patients with HF and AF. Our study highlights the importance of HR control in this specific patient population, as patients with an HR >81 beats/min at discharge had an increased risk of death from any cause and rehospitalization due to HF. Although previous studies reported the effectiveness of lenient HR control, our study interestingly showed that strict HR control led to better prognosis in hospitalized patients with HF and AF.

The Central Illustration in our study demonstrated a linear correlation between HR and the primary endpoint. Analysis of the frequency of the primary endpoint within each HR subgroup yielded the following results: 43.8% in those with HR of 61 to 70 beats/min, 35.6% in patients with HR of 71 to 80 beats/min, and 46.2% in patients with HR exceeding 81 beats/min. It is important to consider that the potential influence of a smaller sample size may have contributed to these observations. Nevertheless, the study's findings are significant, as they established HR of 60 beats/min as a reference value and conducted a comparative analysis between patients with HR >81 beats/min and those with lower HR. These results underscore the importance of controlling HR to a level below 80 beats/min at discharge in this patient population. The study also examined the role of beta-blockers, a common intervention for HR reduction. In the cohort with HR >81 beats/min, 80% were on beta-blocker therapy. Despite this, patients with an HR >81 beats/min exhibited an unfavorable prognosis, raising questions about the reasons behind this observation. One possible explanation could be the difficulties in achieving optimal uptitration of beta-blockers during hospitalization. Furthermore, it is plausible that some patients might have increased their beta-blocker dosage postdischarge. However, considering the study’s focus on HR and medication dynamics during the hospitalization period, monitoring medication regimens following discharge presents a significant challenge. This limitation highlights the complexity of managing HR in patients with HF and AF, especially in the context of medication adjustments and their long-term implications. The study’s findings emphasize the necessity of a comprehensive approach to HR management, including careful consideration of medication titration both during and after hospitalization. Despite guidelines recommending different medical therapies for HFrEF and HFpEF, our study demonstrated that the optimal target HR at discharge was not different between these groups. A previous study showed that HR >81 beats/min was associated with a significantly higher risk of death from any cause in patients with HFrEF but did not focus on HFpEF.^12^ The prescription of beta-blockers was recommended for improving prognosis in patients with HFrEF and for HR control in patients with AF.^13^^,^^14^ We also analyzed beta-blocker users and found that maintaining HR below 80 beats/min significantly enhanced outcomes. Therefore, alongside traditional medical treatments, HR management is crucial in treating HF. The study cohort comprised elderly individuals, a crucial demographic to consider in medical research. During the initial 30-day follow-up, 10 patients succumbed to various causes. Notably, cardiovascular events accounted for the majority of deaths (5 cases), followed by sepsis (1 case), cancer (2 cases), and two cases of unknown etiology. This predominance of cardiovascular mortality underscores its significance in this population. Importantly, adverse events specifically linked to advanced age did not exhibit a significant association in this investigation. The beneficial effects of HR control in HF and AF may involve the reduction of sympathetic tone activation, which plays an important role in the progression of acute HF.^15^ HR control suggests a reduction in sympathetic tone, leading to a favorable prognosis and highlighting the benefits of HR reduction. Braunwald reported that HR reduction decreases myocardial oxygen demand and increases diastolic perfusion time.^16^ This extension in diastolic perfusion time increases coronary flow.^17^ Although an increased HR at admission may be a physiological compensatory mechanism, tachycardia itself can cause HF in AF. As a result, HR control in patients with HF and AF may be effective for prognosis after discharge.

The debate between rhythm control and rate control in the treatment of persistent AF was controversial. Recent studies showed that early rhythm control therapy demonstrated superiority over rate control therapy in patients with HF and AF.^18^^,^^19^ However, catheter ablation is not a treatment indication for all hospitalized patients with HF, especially during the acute phase. Rate control remains a valuable interim therapy between discharge and catheter ablation. Therefore, maintaining a HR <80 beats/min at the time of discharge can provide significant benefits, ensuring patient safety during the period leading up to catheter ablation.

### Study Limitations

First, it was a retrospective study performed in a single center, which resulted in a relatively small sample size. This may limit the generalizability of our findings to other populations and settings. Second, the exclusion of a significant number of patients from the initial database resulted in a smaller but more focused cohort. As a result, the findings of this study are specific to patients with persistent AF during hospitalization and do not extend to those with paroxysmal AF or other forms of heart rhythm irregularities. This selective approach, while beneficial for achieving the study's objectives, does limit the generalizability of the results. Third, we did not evaluate the echocardiography results during follow-up visits, which could potentially influence the prognoses of the patients. Fourth, we did not have detailed information on the specific medication doses, adherence, or adjustments made during the follow-up period, which could also affect patients' outcomes. Fifth, the study did not employ a detailed classification system to distinguish between these subtypes of AF, which represents a limitation in the granularity of our data, and we did not monitor the transition from AF to sinus rhythm in the patient cohort postdischarge, which restricts our understanding of the patients' rhythm status over time. This is a significant consideration, especially in light of subsequent studies highlighting the benefits of rhythm control in AF patients. Additionally, while our findings suggest no major adverse effects in terms of mortality and rehospitalization rates within this HR group, the lack of data on bradycardia-related symptoms represents a gap in our study. Finally, as with any observational study, the possibility of residual confounding cannot be ruled out despite adjusting for multiple potential confounders in our analyses. There was no data on variables such as smoking, physical activity, and alcohol consumption. These variables possessed the potential to exert notable influence on HR. Given the limitations of our study, further prospective investigations with larger samples and more comprehensive data collection are needed to confirm our findings and determine the optimal management strategy for patients with HF and AF.

## Conclusions

The present study underscores the importance of an optimal target HR at discharge for improving prognosis in patients with HF and AF. The prognostically optimal HR at discharge was determined to be <80 beats/min, providing significant clinical benefits across various patient subgroups. These findings highlight the need for a more individualized approach to HR control in patients with HF and AF, taking into account their clinical characteristics and risk factors. Further research is needed to confirm our findings and determine the optimal HR control strategy in different HF phenotypes and in the context of various therapeutic interventions.

PERSPECTIVES **COMPETENCY IN MEDICAL KNOWLEDGE:** Optimal HR control (≤80 beats/min) during hospitalization was associated with better outcomes in patients with HF and AF, offering a valuable therapeutic strategy to enhance prognosis and reduce adverse events.

**TRANSLATIONAL OUTLOOK:** HR control of ≤80 beats/min in patients with HF and AF leads to better clinical outcomes after discharge. To determine if HR management improves long-term outcomes, prospective, large-scale, randomized trials are needed.

## Funding support and author disclosures

The research presented in this manuscript was conducted at Tokyo Women’s Medical University, 8-1 Kawada-cho, Shinjuku-ku, Tokyo, Japan. The authors have reported that they have no relationships relevant to the contents of this paper to disclose.
