# Supplementary material for: Prognostically Optimal Heart Rate at Discharge in Hospitalized Patients With Heart Failure and Atrial Fibrillation
Source: JACC Adv. 2024 Jul 24;3(8):101120. doi: 10.1016/j.jacadv.2024.101120 (PMC11327451; doi:10.1016/j.jacadv.2024.101120)
Supplement: Supplemental material [file mmc1.docx]

**Supplemental Figure 1. Patient tree**

AF = atrial fibrillation; HF = heart failure; HR = heart rate.

**Supplemental Table 1.** Hazard ratios of all-cause mortality and hospitalization for heart failure according to heart rate levels among beta-blocker medication users (N = 255)

|  | **Heart rate, beats/min** | | | |
| --- | --- | --- | --- | --- |
|  | **≤ 60** | **61–70** | **71–80** | **≥ 81** |
| Patient numbers | 52 | 67 | 84 | 52 |
| Person-days | 36,638 | 44,579 | 46,000 | 17,407 |
| Cases | 18 | 28 | 27 | 25 |
| Rate/100,000 person-days | 49 | 63 | 59 | 144 |
| Model 1 | 1.00 (reference) | 1.27 (0.70–2.31) | 1.15 (0.63–2.09) | 2.34 (1.26–4.34) |
| Model 2 | 1.00 (reference) | 1.30 (0.71–2.36) | 1.17 (0.64–2.13) | 2.32 (1.24–4.34) |
| Model 3^a^ | 1.00 (reference) | 1.25 (0.68–2.31) | 1.11 (0.60–2.04) | 2.26 (1.19–4.27) |

Model 1: adjusted for age and sex.

Model 2: adjusted for model 1 covariates plus LVEF, BNP, eGFR, and RASi medication.
Model 3: adjusted for model 2 covariates plus BMI, systolic blood pressure, and diabetes.

^a^The number of overall patients was 246.

BMI = body mass index; BNP = brain natriuretic peptide; eGFR = estimated glomerular filtration rate; LVEF = left ventricle ejection fraction; RASi = renin-angiotensin-aldosterone system inhibitor.

**Supplemental Table 2.** Hazard ratios of all-cause mortality and hospitalization for heart failure according to heart rate levels, after excluding participants who had all-cause mortality and hospitalization for heart failure during initial 30 days follow-up (N = 320)

|  | **Heart rate, beats/min** | | | |
| --- | --- | --- | --- | --- |
|  | **≤ 60** | **61–70** | **71–80** | **≥ 81** |
| Patient numbers | 78 | 84 | 97 | 61 |
| Person-days | 47,987 | 52,703 | 52,087 | 22,962 |
| Cases | 27 | 34 | 32 | 26 |
| Rate/100,000 person-days | 56 | 65 | 61 | 113 |
| Model 1 | 1.00 (reference) | 1.14 (0.69–1.90) | 1.09 (0.65–1.83) | 1.85 (1.07–3.19) |
| Model 2 | 1.00 (reference) | 1.19 (0.71–1.98) | 1.18 (0.70–2.00) | 1.76 (1.02–3.05) |
| Model 3^a^ | 1.00 (reference) | 1.11 (0.66–1.87) | 1.07 (0.63–1.82) | 1.74 (0.99–3.07) |

Model 1: adjusted for age and sex.

Model 2: adjusted for model 1 covariates plus LVEF, BNP, eGFR, beta-blocker medication, and RASi medication.
Model 3: adjusted for model 2 covariates plus BMI, systolic blood pressure, and diabetes.

^a^The number of overall patients was 306.BMI = body mass index; BNP = brain natriuretic peptide; eGFR = estimated glomerular filtration rate; LVEF = left ventricle ejection fraction; RASi = renin-angiotensin-aldosterone system inhibitor.

**Supplemental Table 3.** Hazard ratios of all-cause mortality and hospitalization for heart failure according to heart rate levels in the subgroup analyses by LVEF (N = 334)

|  | **Heart rate, beats/min** | | | |
| --- | --- | --- | --- | --- |
|  | **≤ 60** | **61–70** | **71–80** | **≥ 81** |
| **LVEF < 50% (N = 196)** | | | | |
| Patient numbers | 41 | 61 | 54 | 40 |
| Person-days | 24,128 | 39,113 | 30,529 | 13,981 |
| Cases | 15 | 26 | 20 | 20 |
| Rate/100,000 person-days | 62 | 66 | 66 | 143 |
| Model 1 | 1.00 (reference) | 0.98 (0.52–1.85) | 1.05 (0.54–2.07) | 1.71 (0.86–3.39) |
| Model 2 | 1.00 (reference) | 0.97 (0.51–1.86) | 1.14 (0.58–2.25) | 1.67 (0.83–3.33) |
| Model 3^a^ | 1.00 (reference) | 0.91 (0.47–1.77) | 0.98 (0.49–1.97) | 1.60 (0.79–3.24) |
| **LVEF ≥ 50% (N = 138)** | | | | |
| Patient numbers | 38 | 28 | 47 | 25 |
| Person-days | 23,876 | 13,684 | 21,638 | 8,995 |
| Cases | 13 | 13 | 16 | 10 |
| Rate/100,000 person-days | 54 | 95 | 74 | 111 |
| Model 1 | 1.00 (reference) | 1.68 (0.76–3.71) | 1.34 (0.63–2.84) | 2.08 (0.88–4.90) |
| Model 2 | 1.00 (reference) | 1.79 (0.79–4.06) | 1.36 (0.61–3.00) | 1.90 (0.78–4.62) |
| Model 3^b^ | 1.00 (reference) | 1.60 (0.69–3.70) | 1.25 (0.57–2.77) | 1.67 (0.64–4.34) |

Model 1: adjusted for age and sex.

Model 2: adjusted for model 1 covariates plus LVEF, BNP, eGFR, beta-blocker medication, and RASi medication.

Model 3: adjusted for model 2 covariates plus BMI, systolic blood pressure, and diabetes.

^a^The number of overall patients was 189.

^b^The number of overall patients was 129.

There was no significant interaction between resting heart rate category (4 group) and LVEF category (2 group) (*P* for interaction = 0.893).

BMI = body mass index; BNP = brain natriuretic peptide; eGFR = estimated glomerular filtration rate; LVEF = left ventricle ejection fraction; RASi = renin-angiotensin-aldosterone system inhibitor.

**Supplemental Table 4.** Hazard ratios of all-cause mortality or hospitalization for heart failure according to heart rate levels (N = 334)

|  | **Heart rate, beats/min** | | | |
| --- | --- | --- | --- | --- |
|  | **≤ 60** | **61–70** | **71–80** | **≥ 81** |
| **All-cause mortality** | | | | |
| Patient numbers | 79 | 89 | 101 | 65 |
| Person-days | 54,868 | 72,253 | 58,405 | 28,717 |
| Cases | 12 | 11 | 11 | 19 |
| Rate/100,000 person-days | 22 | 15 | 19 | 66 |
| Model 1 | 1.00 (reference) | 0.73 (0.32–1.66) | 0.85 (0.37–1.93) | 2.89 (1.38–6.07) |
| Model 2 | 1.00 (reference) | 0.60 (0.26–1.42) | 0.88 (0.38–2.02) | 2.68 (1.25–5.76) |
| Model 3^a^ | 1.00 (reference) | 0.42 (0.17–1.06) | 0.65 (0.27–1.58) | 1.93 (0.86–4.36) |
| **Hospitalization for heart failure** | | | | |
| Patient numbers | 79 | 89 | 101 | 65 |
| Person-days | 48,537 | 52,797 | 53,221 | 22,976 |
| Cases | 22 | 34 | 28 | 17 |
| Rate/100,000 person-days | 45 | 64 | 53 | 74 |
| Model 1 | 1.00 (reference) | 1.39 (0.81–2.38) | 1.16 (0.66–2.04) | 1.49 (0.78–2.82) |
| Model 2 | 1.00 (reference) | 1.45 (0.84–2.51) | 1.28 (0.73–2.27) | 1.46 (0.77–2.78) |
| Model 3^a^ | 1.00 (reference) | 1.35 (0.78–2.34) | 1.16 (0.65–2.05) | 1.51 (0.79–2.89) |

Model 1: adjusted for age and sex.

Model 2: adjusted for model 1 covariates plus LVEF, BNP, eGFR, beta-blocker medication, and RASi medication.

Model 3: adjusted for model 2 covariates plus BMI, systolic blood pressure, and diabetes.

^a^The number of overall patients was 318.

BMI = body mass index; BNP = brain natriuretic peptide; eGFR = estimated glomerular filtration rate; LVEF = left ventricle ejection fraction; RASi = renin-angiotensin-aldosterone system inhibitor.
